# Supplementary figures and images for: Sugar inhibits brassinosteroid signaling by enhancing BIN2 phosphorylation of BZR1
Source: PLoS Genet. 2021 May 14;17(5):e1009540. doi: 10.1371/journal.pgen.1009540 (PMC8153450; doi:10.1371/journal.pgen.1009540)

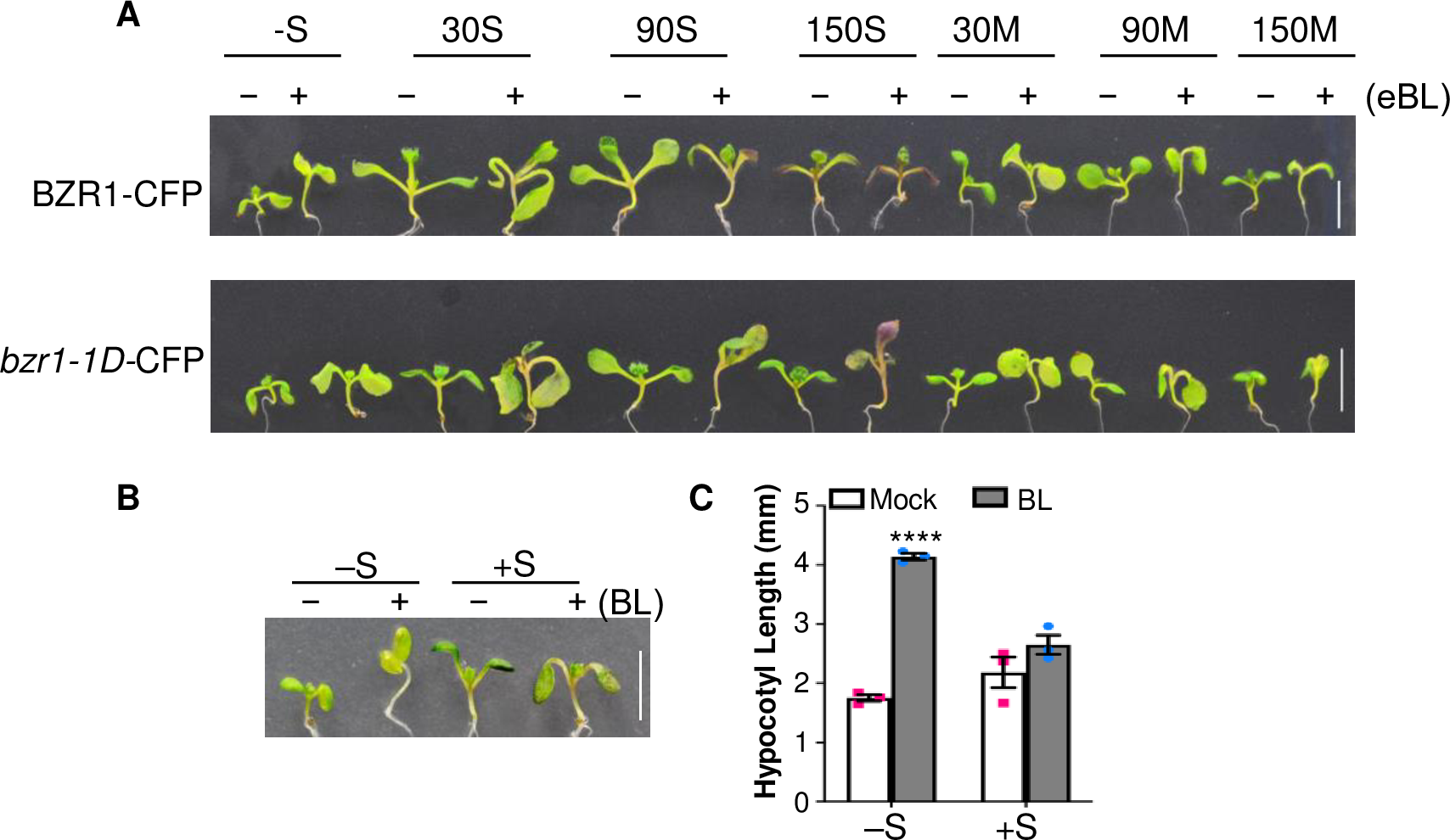

Supplement: S1 Fig — (A) Phenotypes of BZR1-CFP and bzr1-1D-CFP grown on media containing no sugar (-S), 30 to 150 mM sucrose (S) or mannitol (M), as in Fig 1. Bar = 5 mm. (B) Phenotypes of Col-0 grown on medium containing 0 (-BL) or 10 nM brassinolide (+BL) and 0 (-S) or 90 mM sucrose (+S) for six days. (C) Hypocotyl length of seedlings shown in panel B. Error bars indicate the standard error of the mean (SEM, three independent experiments, n≥ 20). **** P<0.0001 (Student’s t test). (TIF) [file pgen.1009540.s001.tif]

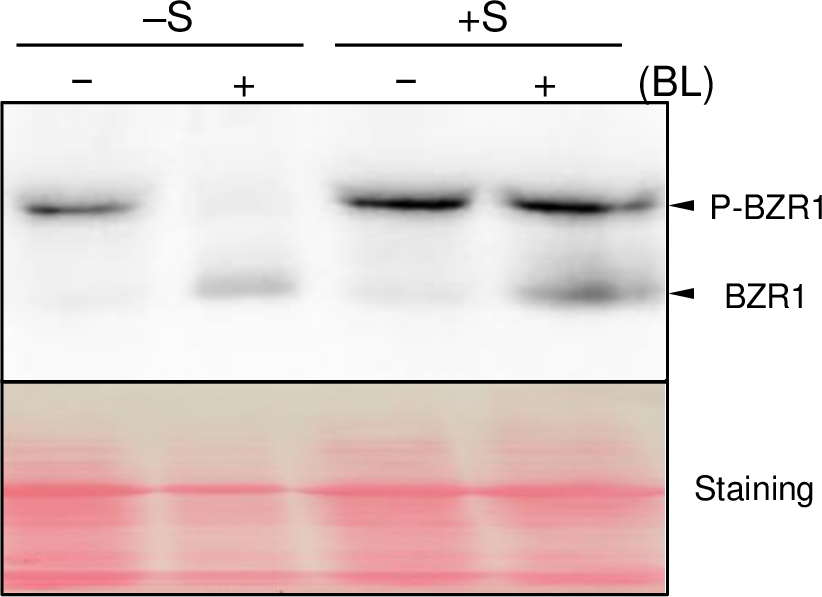

Supplement: S2 Fig — Immunoblot analysis of BZR1 protein with BZR1 antibody in Col-0 grown on medium containing 0 (-BL) or 10 nM brassinolide (+BL) and 0 (-S) or 90 mM sucrose (+S) for six days. (TIF) [file pgen.1009540.s002.tif]

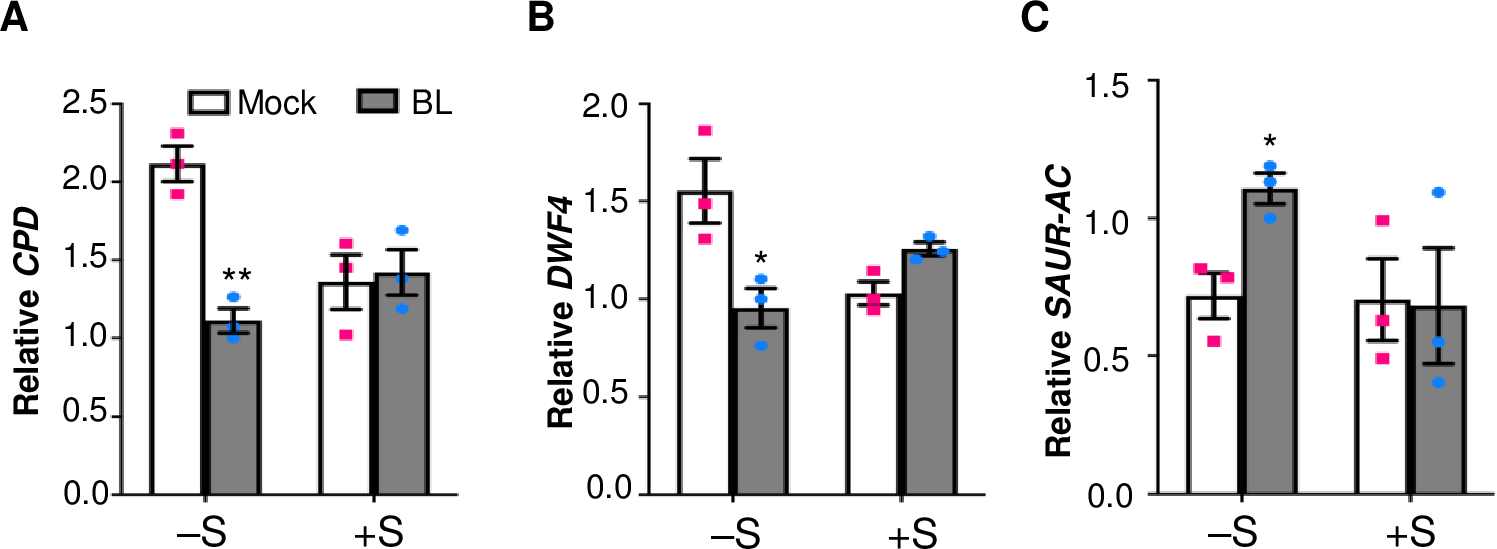

Supplement: S3 Fig — (A-C) Relative expression of CPD, DWF4 or SAUR-AC analyzed by qRT-PCR in Col-0 seedlings grown on medium containing 0 (-BL) or 10 nM brassinolide (+BL) and 0 (-S) or 90 mM (+S) sucrose for six days. Error bars indicate the SEM (three independent experiments). ** P< 0.01, * P<0.05 (Student’s t test). (TIF) [file pgen.1009540.s003.tif]

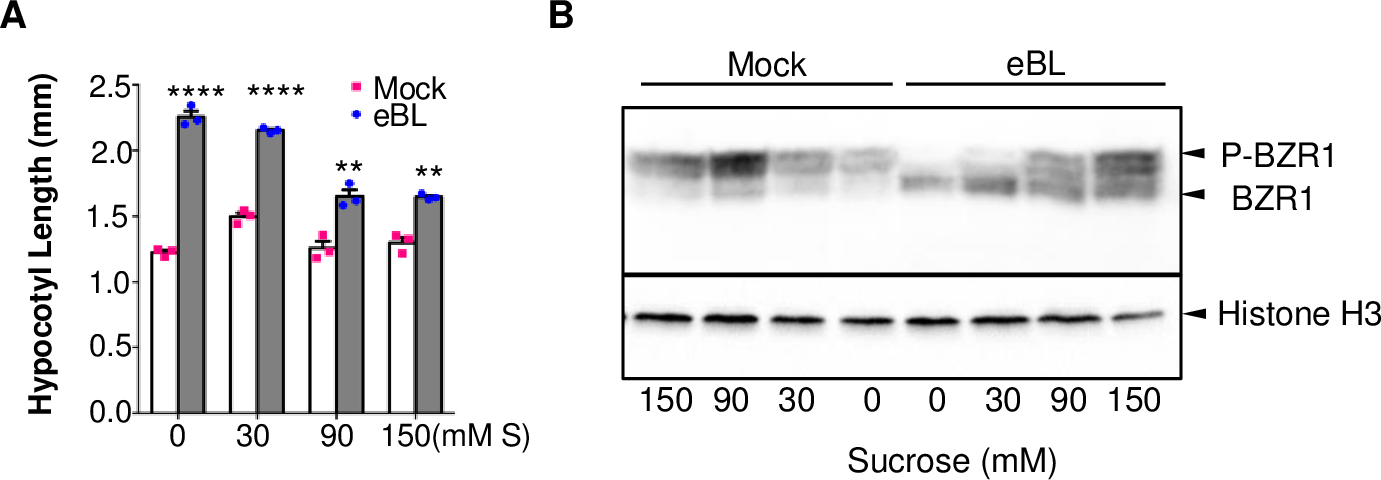

Supplement: S4 Fig — (A) Hypocotyl length of BZR1S173A-CFP/Col-0 grown on medium containing 100 nM eBL and indicated concentrations of sucrose. (B) Immunoblot analysis of BZR1S173A-CFP protein in seedlings in (A). Histone H3 was probed as a loading control in the immunoblot analysis. (TIF) [file pgen.1009540.s004.tif]

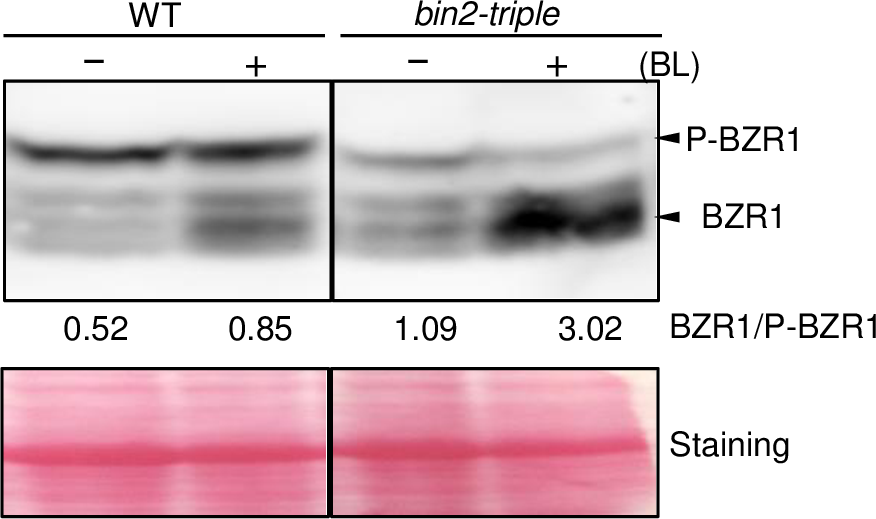

Supplement: S5 Fig — Immunoblot analysis of BZR1 protein in wild type and the bin2-triple mutant grown on media containing 90 mM sucrose and 0 (-BL) or 10 nM BL (+BL) for six days. The ratio of dephosphorylated BZR1 to phosphorylated BZR1 (BZR1/P-BZR1) was measured. (TIF) [file pgen.1009540.s005.tif]
