# Supplementary material for: Sugar inhibits brassinosteroid signaling by enhancing BIN2 phosphorylation of BZR1
Source: PLoS Genet. 2021 May 14;17(5):e1009540. doi: 10.1371/journal.pgen.1009540 (PMC8153450; doi:10.1371/journal.pgen.1009540)
Supplement: Attachment — Submitted filename: Response to Reviewer comments-zw.docx [file pgen.1009540.s008.docx]

Dear editor,

Thank you very much for your handling our manuscript and helpful comments on revising our work. We have carefully revised the manuscript to address your comments and those of reviewers. Specifically, we have made the following changes and improvements.

1. In Fig 3 and S1 Fig, we added phenotype images.

2. In S2 Fig, we added an immunoblot of BZR1 protein in wild type Col-0.

3. In S3 Fig, we added qRT-PCR analysis of sugar effects on BZR1 target gene expression.

4. In S5 Fig, we added immunoblot data showing BR induced BZR1 dephosphorylation in the *bin2-triple* mutant.

Below are our “Point-by-point responses to reviewers’ comments.

We hope you find our revised manuscript acceptable for publication in ***Plos Genetics****.*

Sincerely yours,

Zhi-Yong Wang

**Point-by-point response to comments from Reviewer**

**Review 1:**

The present manuscript describes that sugar inhibits brassinosteroid (BR) signaling pathway in the light, and BR induction of hypocotyl elongation in seedlings grown under light is inhibited by increasing concentration of sucrose. Through phenotypic analysis and immunoblot analysis, the authors demonstrate that sucrose inhibits the BR-induced hypocotyl elongation and dephosphorylation of BZR1 in light. Furthermore, the authors find that high sucrose inhibits BR-induced dephosphorylation of BZR1. Moreover, the authors present that sucrose inhibition of BR signaling in light dependents on the regulation of BIN2. This work illustrates an intricate three-way crosstalk whereby the combination of light and sugar signals modulate the BR signaling pathway to optimize growth according to both environmental and metabolic conditions, which is of novelty and interests to researchers in the fields of light, phytohormone and sugar signaling. However, the authors need to address the comments and concerns I raised below.

**Response:**

Thank you! We appreciate your comments on the novelty and broad interest of our work.

**Comment 1** The authors used BZR1-CFP and bzr1-1D-CFP to analyze the hypocotyl lengths (Figure 1), but did not include wild type or bzr1 /mutant or BZR1-RNAi plants. These data are not sufficient to support the authors’ conclusion that sucrose inhibits the BR-induced hypocotyl elongation and dephosphorylation of BZR1. The authors should perform these analyses again with at least wild type plants.

**Response to Comment 1:**

Thanks for this helpful comment. We analyzed the hypocotyl elongation of Col-0 and bzr1-1D after sucrose/glucose and BL treatments in Fig.4A-4B, which showed similar response as BZR1-CFP and bzr1-1D-CFP. We also have performed the same experiments with wild type (Col-0), and added data of hypocotyl lengths (S1B and S1C Fig) and BZR1 phosphorylation (S2 Fig). The results are consistent with those using BZR1-CFP which shows no phenotypic difference from wild type.

We did not include the mutant of bzr1 because it shows no phenotype (due to redundancy with its homologs).

**Comment 2：**As shown in Figures 2 A and C, as sucrose concentration increases, the protein levels of P-BZR1 and BZR1 increase accordingly, and P-BZR1 and BZR1 proteins are not at the similar levels at the very beginning. Therefore, it’s not appropriate to conclude that high sucrose inhibits BR-induced dephosphorylation of BZR1. Please clarify this complication.

**Response to Comment 2:**

We quantified the total BZR1 protein level and added the data to Fig 2A and C. While the total BZR1 protein level is about 80% higher in +S than -S sample without BR treatment, it reached similar level after BR treatment when the BZR1/P-BZR1 ratio (measurement of BZR1 dephosphorylation) is lower under +S than -S. In other words, when BR is present at 10 to 1000 nM, the total BZR1 protein level is similar but more BZR1 is dephosphorylated in the -S sample than the +S sample. Therefore, the result does indicate that sugar inhibits BR-induced BZR1 dephosphorylation.

**Comment 3：** The authors stated that high concentration of sucrose inhibits BR-induced dephosphorylation of BZR1. Unfortunately, the authors did not detect the protein levels of P-BZR1 and BZR1 in bin2-triple mutant, which is important to support their conclusion.

**Response to Comment 3:**

Following your suggestion, we analyzed the BZR1 protein in the *bin2-triple* mutant and added the results in S5 Fig. BL treatment caused a bigger change of BZR1 phosphorylation in the *bin2-triple* mutant (3x) than in wild type (≤2x) in high sugar condition, supporting our conclusion.

**Comment 4:** The authors conclude that sucrose inhibits BR signaling independent of 14-3-3 proteins, HXK1 and TOR. But the authors did not explain how high concentration of sucrose may inhibit BR signaling. Please discuss the possible molecular mechanisms. Moreover, the results showing that sucrose inhibition of BR signaling is independent of HXK1 and TOR should be included in the supplemental materials.

**Response to Comment 4:**

We discussed the possible molecular mechanisms, such as RGS, O-GlcNAc and O-fucose, or other unknown mechanisms. Identification of the molecular mechanism that mediates sugar inhibition of BR signaling is important but will require significant effort. Considering the importance of HXK1 and TOR in sugar crosstalk with hormonal pathways, we believe the results should be shown in Fig 4 not supporting information, so that readers who question the conclusion can see the results easily.

**Comment 5:** In Figures 1F and 3B, the loading control protein histone H3 is not at the same level.

**Response to Comment 5:**

We agree the loading was not perfect. However, the histone H3 serves as a reference and the key information was the ratio difference between P-BZR1 and BZR1 forms not the abundance of BZR1.

**Comment 6:** In Figure S1B, “Mock” and “eBL” are not located at the central of the line. “Histone H3” does not show the band correctly. The similar problems are found in Figures 1, 2, 3, and 4.

**Response to Comment 6:**

We have corrected these errors.

**Comment 7:** The data shown in Figures 3C and 3D should be analyzed by student’s t test.

**Response to Comment 7:**

We analyzed the data of Figure 3C by student’s t test as you suggested. After analyzing the *p* value in Fig 3C, it is obvious enough to see the differences of seedlings after BL treatment in different sugar conditions， so we did not use the relative elongation data in original Fig 3D any more.

**Comment 8:** Given the authors’ conclusion that sucrose inhibits BR signaling independent of HXK1 and TOR, it appears that the trend of BZR1 phosphorylation in BZR1-CFP/Col-0 and the BZR1-CFP/gin2-1 seedlings shown in Figure 4C is not consistent with this conclusion. Please clarify.

**Response to Comment 8:**

We are not sure why you think the result is not consistent with our conclusion. Figure 4C shows that in both Col-0 and *gin2-1*, BR induced complete dephosphorylation of BZR1 in the absence of sugar but only partial dephosphorylation in the presence of sugar. In other words, sugar inhibited BR-induced BZR1 dephosphorylation in *gin2-1*, thus independent of HXK1.

**Reviewer #2:**

Brassinosteroids (BRs) are a family of plant steroid hormones that play crucial roles throughout plant growth and development. Through BRI receptor, BR signal inhibits BIN2 kinase and results in dephosphorylation and activation of the master transcription factors BZR1 and BES1 to regulate thousands of BR target genes. In dark, BR promotes hypocotyl growth, while under light, BR inhibit hypocotyl elongation. It was shown that sugar-TOR signaling promote hypocotyl growth by stabilizing BZR1 protein when light grown seedlings shift to darkness (ref1). However, the mechanism of how BR inhibit seedling hypocotyl elongation is still unclear. The authors reported an interesting finding that under light high sucrose inhibits BR signaling by stabilizing BIN2 kinase and therefore increasing BZR1 phosphorylation and degradation/inactivation. This paper connects BR signaling with nutrient status and provides interesting observation that BIN2 and BZR1 protein levels correlate with sugar inhibition.

**Response:**

Thank you! We appreciate your comments.

**Comment 1**: As hypocotyl length is one measurement of BR/BZR1/BES1 activity, it would be nice to have seedling pics of those various sugar & BL treatments, at least for Fig 1, 2,3. Reference 1 is a good example for combining phenotypes and quantitative measurements.

**Response to Comment 1:**

We added corresponding seedling pictures in S1A Fig and Figure 3C to Figure 1 and Figure 3. Since experiments in Figure 2 are short-term treatment (less than 2 hours) without any difference in phenotype, we did not show the seedling picture for Figure 2.

**Comment 2**: do authors also have other evidence for BZR1 activity, such as target gene expression change by Q-RT-PCR and binding of BZR1 to its target genes by ChIP-PCR? Reference 2 provides RT and ChIP-PCR target genes. The reason is that WB exposure varies from gel to gel, which makes it hard to compare between samples. For example, in fig 4 D and F, the measurements of hypocotyl length are not consistent with the BZR1 band intensity between samples treated w/ and w/o Estradiol.

**Response to Comment 2:**

We appreciate your suggestion and have performed qRT-PCR analysis of BZR1 target genes (*CPD*, *DWF4* and *SAUR-AC*). The results are consistent with our conclusion and are shown in S3 Fig.

**Comment 3**: BIN2 has been reported to be a direct target of TOR-S6K signaling (ref 3). When the authors claimed that the sugar inhibition is independent of TOR, the possibility was not ruled out that high sugar might reduce TOR activity, which releases BIN2 inhibition and results in decreased BZR1 activity.

**Response to Comment 3:**

Thanks for your comments. We modified our discussion: While TOR mediates sugar-dependent stabilization of BZR1 in the dark [[2](https://docs.google.com/document/d/1VpqcpKpxDljlmf531Ts-H8AUH4sHZE9g/edit#heading=h.3znysh7)] and BIN2 has been reported to be direct target of TOR-S6K signaling [3], inducible silencing of TOR made no obvious difference in the sugar effect on BZR1 phosphorylation or hypocotyl elongation responses to BR.

**Comment 4**:
Fig 3E, the label needs to correspond to samples.

**Response to Comment 4:**

We corrected these errors.

**Comment 5**: In some figures, such as Fig1D and E, western exposure time varies a lot that makes the similar samples look so different (for example, Suc 0, eBL Vs Mannitol 0, eBL)

**Response to Comment 5:**

The exposure time and image contrast were adjusted to have the best visual effect for each individual image. Raw signals should not be compared directly between gels.

**Reviewer 3**

Light, sugar, and hormones are three major signals to regulate plant growth and development. In this report, the authors found that under a light condition, high concentrations of sugar can inhibit BR signaling via enhancing the activity of BIN2 and preventing the accumulation of unphosphorylated BES1 upon the treatment of BL, the most active BR. As a result, the hypocotyl elongation is inhibited. Such a physiological effect relies on the functional BIN2 but is independent of known sugar sensors, Hexokinase 1 (HXK1) and Target of Rapamycin (TOR). Generally speaking, the data are solid and interesting. There are a few suggestions to improve the manuscript.

**Response:**

Thank you! We appreciate your comments.

**Comment 1**: The authors suggest that such an effect does not depend on HXK1 or TOR signaling pathway. What kind of molecular mechanisms are possibly involved in? The authors should at least discuss a number of possibilities. It seems they identified something interesting but did not dig deep enough to elucidate mechanisms beyond the data themselves.

**Response to Comment 1:**

Thank you for this comment. We added discussion of possible molecular mechanisms involving receptors on plasma membrane (RGS) and O-GlcNAc/O-fucose modifications.

**Comment 2**: The authors should include loss of function mutants of BES1 and homologs in their analyses. Such mutants have been isolated and reported in literature.

**Response to Comment 2:**

The mutants lacking all BZR1 family members are BR insensitive (like bri1) and thus not useful for studying mechanisms that modulate BR sensitivity, whereas loss of a single member has little phenotypic effect due to genetic redundancy.

**Comment 3**:	Why did the authors use 90 and 150 mM sucrose in their experiments? How much endogenous sucrose is there in Arabidopsis under a light condition?

**Response to Comment 3:**

Many published studies used 90 mM (3%) sucrose. The effect of sucrose is dose dependent， and a weak effect was observed at 30 mM sucrose. The endogenous sucrose level is likely variable depending on synthesis, transport, and uptake.

**Comment 4**:	The sentence in lines 82-84 should be altered. Inactivation of BIN2 cannot result in the dephosphorylation of BZR1 by protein phosphatase 2A.

**Response to Comment 4:**

Thank you! We have reorganized this sentence.

**Comment 5**:	In Figure 3E, “-“ and “+” are not aligned well with the columns.

**Response to Comment 5:**

We corrected them in the corresponding figure.
